# Supplementary material for: Generative model for the first cell fate bifurcation in mammalian development
Source: Development. 2025 Sep 5;152(17):dev204717. doi: 10.1242/dev.204717 (PMC12327801; doi:10.1242/dev.204717)
Supplement: Supplementary information [file develop-152-204717-s1.pdf]

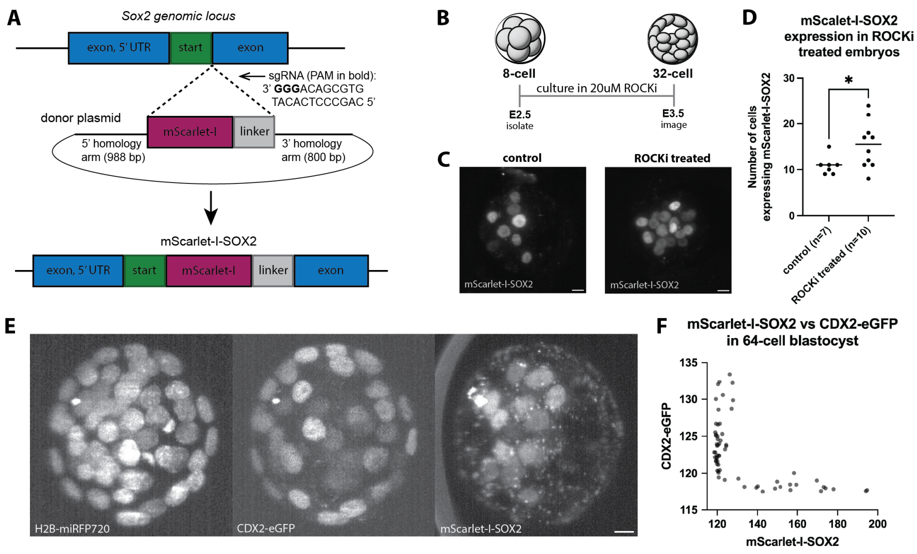

Fig. S1. A) Targeting strategy for *mScarlet-I-Sox2* reporter mouse line. *mScarlet-I* plus a linker sequence was targeted to the N-terminus of the *Sox2* gene. B) Method for validation of *mScarlet-I-Sox2* reporter mouse line. 8-cell (E2.5) embryos were isolated and treated with 20μM ROCKi until the 32-cell stage (E3.5). At E3.5, ROCKi-treated embryos were live imaged for mScarlet-I-SOX2 alongside controls. C) Sample images of control vs. ROCKi-treated embryos, representative of $N = 7$ control, $N = 10$ ROCKi-treated embryos. Scale bar: 10μm. D) Quantification of the number of mScarlet-I-SOX2 expressing cells in control embryos versus embryos treated with 20μM ROCKi. In agreement with expected SOX2 behavior, embryos treated with ROCKi displayed significantly higher numbers of SOX2-expressing cells. Each point represents one embryo (Student's t-test, $p = 0.0432$). E) Live images of a 64-cell stage embryo expressing H2B-miRFP670 (nuclei), CDX2-eGFP, and mScarlet-I-SOX2. Scale bar: 10μm. F) Quantification of cells' expression levels of mScarlet-I-SOX2 versus CDX2-eGFP for the 64-cell stage blastocyst shown in panel (E). Each point represents one cell.

Development • Supplementary information

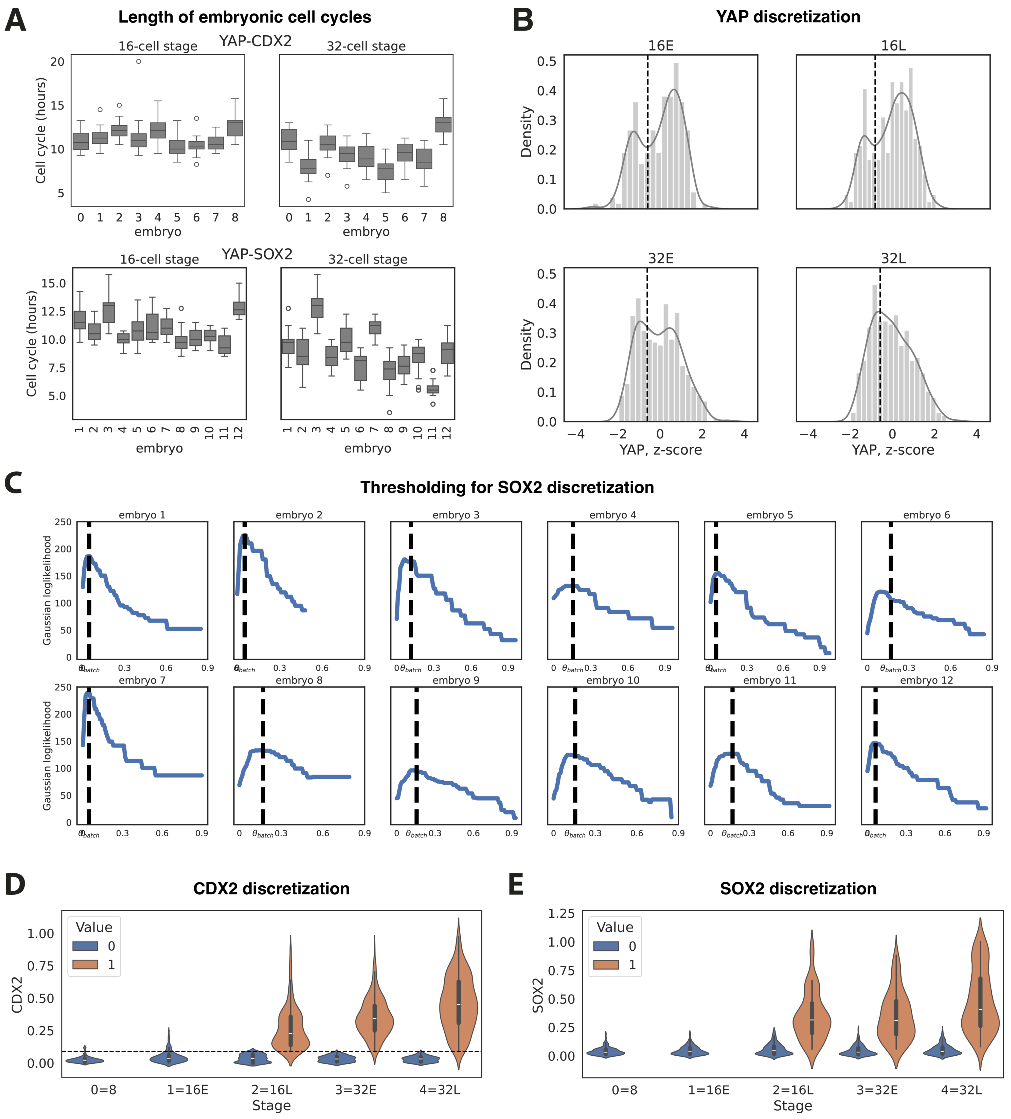

Fig. S2. Data processing and discretization A) Boxplots summarizing the distributions of the cell cycle lengths in the embryos used in this study. x-axis: embryo, y-axis: cell cycle length, units are hours. Top row: YAP-CDX2 data, bottom row: YAP-SOX2 data. B) Histograms showing the distribution of summarized YAP values for every stage (Methods) in the embryos used in this paper. Black vertical dashed line: stage-dependent threshold, adopted from [43]. C) Thresholding procedure for YAP-SOX2 dataset. Due to batch-dependent differences in the background SOX2 intensity, batch-specific thresholds $\theta batch$ were selected to discretize SOX2. Every subplot corresponds to one of 12 YAP-SOX2 embryos. x-axis: candidate threshold. y-axis: corresponding Gaussian loglikelihood (Methods). Black vertical dashed line: $\theta batch$ for the corresponding batch. D) Violinplots showing the distribution of summarized CDX2 values (Methods) for every discretized stage. Color: value after binarization. Black horizontal dashed line: stage-independent threshold. E) Same as C) for SOX2. No threshold shown since it was chosen to be batch-dependent.

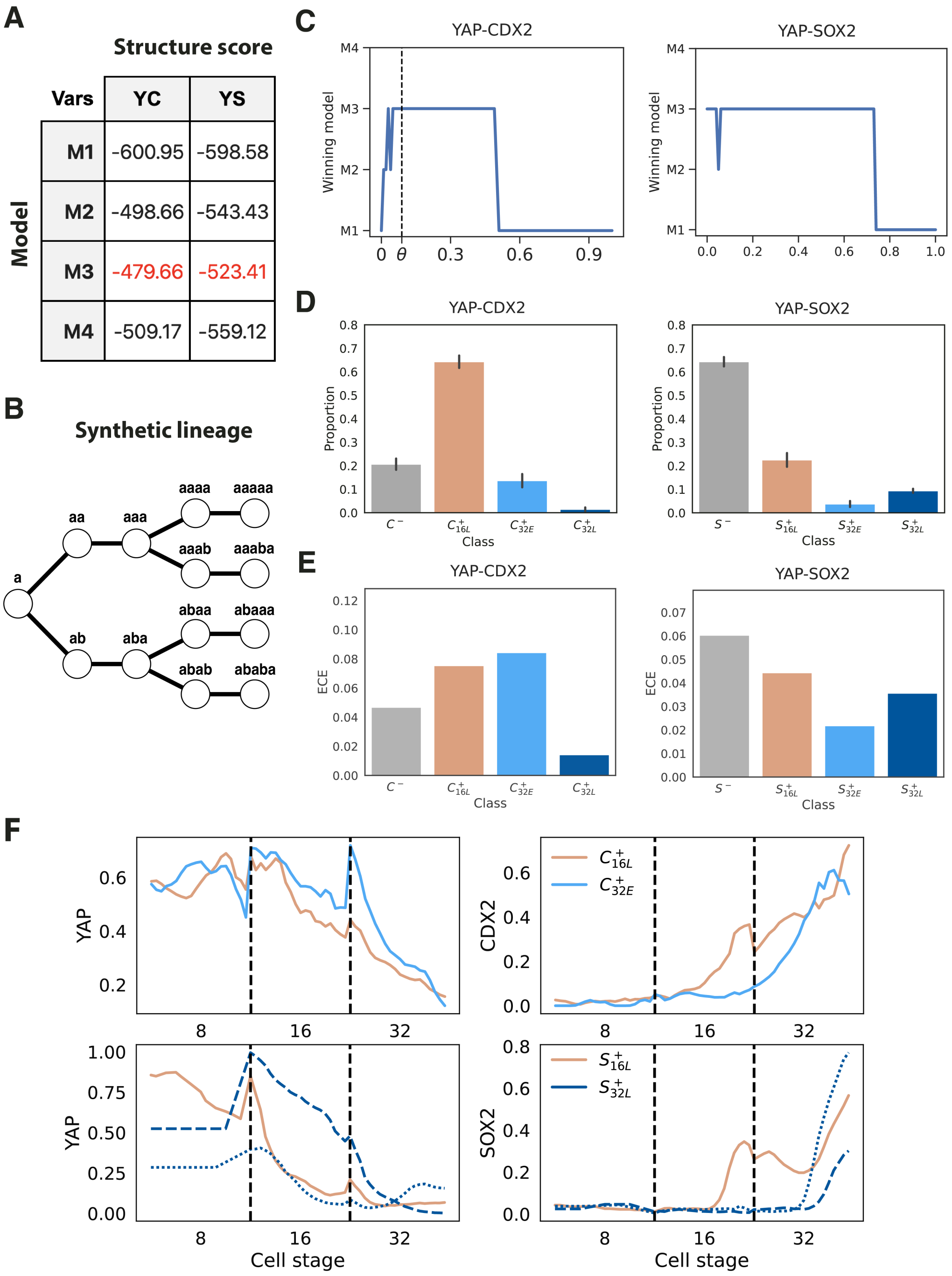
A
Structure score
Vars
YC
YS
Model
M1 -600.95 -598.58
M2 -498.66 -543.43
M3 -479.66 -523.41
M4 -509.17 -559.12
B
Synthetic lineage
a
aa
aaa
aaaa
aaaaa
aaab
aaaba
ab
aba
abaa
abaaa
abab
ababa
C
YAP-CDX2
YAP-SOX2
Winning model
M1
M2
M3
M4
D
Proportion
Class
E
ECE
Class
F
YAP
CDX2
SOX2
Cell stage
8
16
32

Fig. S3. Bayesian model selection and inference details. A) Structure scores (inversely proportional to BIC, see Methods) on training sets for the 4 candidate models from Fig. 3A. Rows: model, Columns: dataset; YC denotes the YAP-CDX2 dataset, YS denotes the YAP-SOX2 dataset. M3 model achieves the best (highest) structure score on the training sets and was therefore selected for modeling both datasets. **B)** A schematic showing an encoding for every node in the synthetic lineages that were simulated from Bayesian networks. The lineages incorporate 2 rounds of divisions over 5 stages. **C)** M3 outperforms other models on the training set over a range of thresholding parameters. For both plots, *x*-axis: thresholding parameter for TF discretization. *y*-axis: winning model. For YAP-CDX2 dataset, black vertical dashed line: selected threshold. Note that for YAPSOX2 dataset, the threshold was selected to be batch-dependent, therefore a universal threshold could not be shown. **D)** Left: predicted frequencies of CDX2 induction classes over all train-test splits (Methods). Bar: mean frequency, error bar: one standard deviation around the mean. Right: same as left for YAP-SOX2 model. **E)** Left: expected calibration error (ECE) for the CDX2 induction class probabilities on held-out test data, mean across all train-test splits. Right: same for the YAP-SOX2 model. **F)** Top: two representative examples of YAP-CDX2 traces demonstrating variable timing of CDX2 induction. Beige: a branch from $C^{+}{}_{16L}$, light blue: a branch from $C^{+}{}_{32E}$. Both branches were classified as nuclear YAP for all stages. Bottom: three representative examples of YAP-SOX2 traces demonstrating variable timing of SOX2 induction and variable corresponding YAP behaviors. Beige: a branch from $S^{+}{}_{16L}$, YAP is lost at 8/16 division for this branch. Dark blue: two branches from $S^{+}{}_{32L}$; dotted line: a branch with YAP lost at 8/16 division, dashed line: another branch with YAP lost at 16/32 division. *x*-axis: warped time, vertical black dashed line marks divisions.

Development • Supplementary information

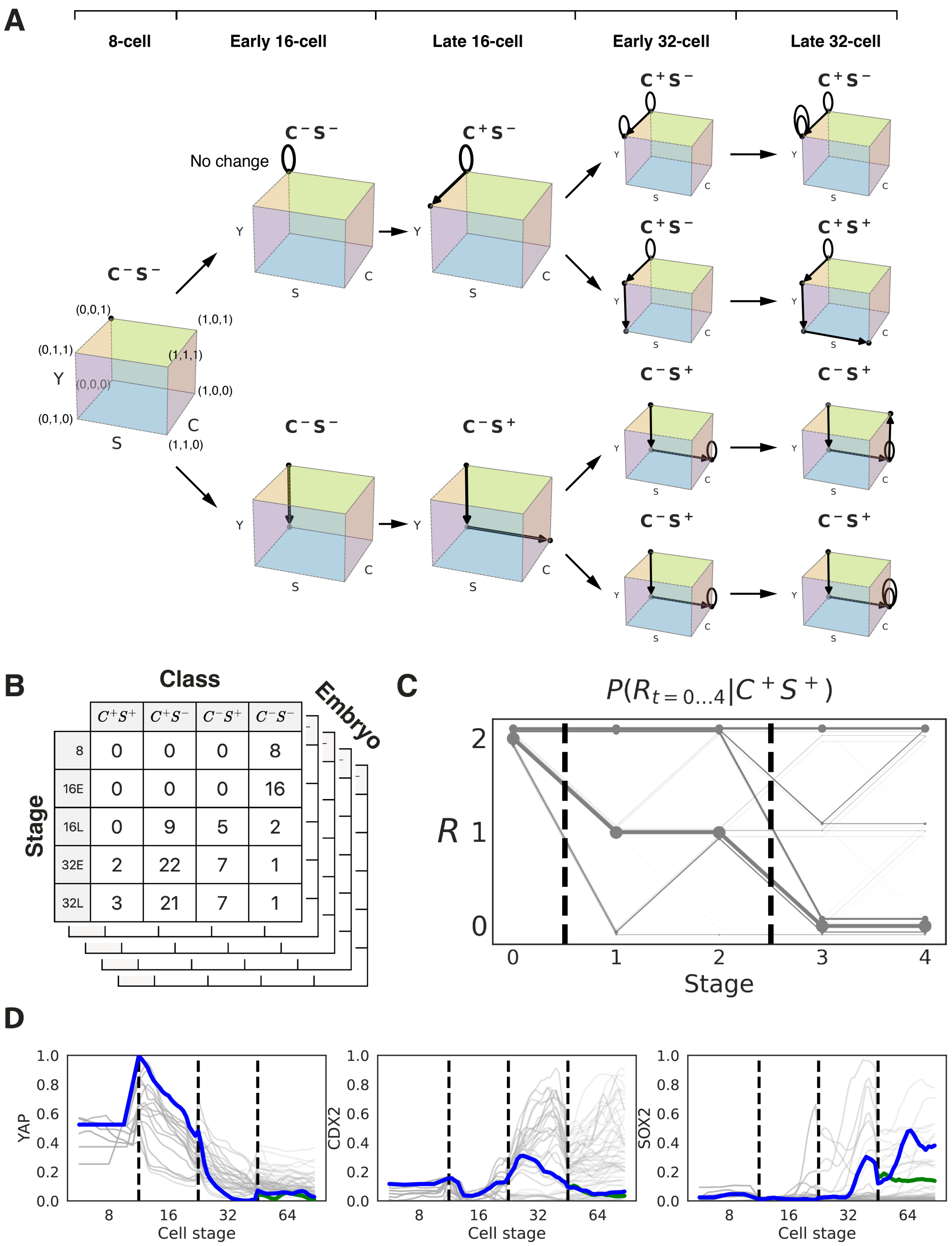

Development • Supplementary information

Fig. S4. Data fusion with Bayesian networks. A) Expression of (S, C, y) in one lineage tree sampled from a fused model. Initially one cell at $(0, 0, 1)$ is sampled. At every step one or two cells are sampled using transition matrices of the fused model. As a result, the trajectory of a cell is a random walk on the $\{0, 1\}^3$ cube, with two branching points resulting in 4 cells at 32L stage. Steps of the walk are shown with arrows. If the sampled value coincides with the previous one, a loop is shown. **(B)** Every simulated embryo is summarized as a table showing numbers of cells in C^+S^+, C^+S^-, C^-S^+, and C^-S^- classes over stages. **(C)** Posterior distribution of relative exposed area R for C^+S^+ cells (i.e., conditioned on $C_4 = 1, S_4 = 1$). R serves as a proxy of cell position: $R = 0$ for inner, $R = 1$ for intermediate, and $R = 2$ for outer cells. Linewidth of a trajectory is proportional to its posterior probability. **(D)** One C^+S^+ cell tracked through 64-cell stage. Both daughters proceed to lose CDX2 expression, with persistent SOX2 expression at 64-cell stage.
